# Supplementary material for: Are carbon emissions trading and green financial instruments synergistic? -Comprehensive quantitative research based on content analysis
Source: PLoS One. 2024 Mar 7;19(3):e0298601. doi: 10.1371/journal.pone.0298601 (PMC10919601; doi:10.1371/journal.pone.0298601)
Supplement: S1 Data — (ZIP) [file pone.0298601.s001.zip › Data/地级市Z_F工作报告.zip!/┴╔─■/╡ñ╢1⁄2╩╨/└┤╫╘╣1⁄2╓┌║┼í╛┬φ┐╦╩2╛▌═°í┐_2013.docx]

各位代表：

　　我代表丹东市人民政府向大会报告工作，请予审议，并请市政协委员和各位列席人员提出意见。

　　一、过去五年主要工作回顾

　　五年来，在省委、省政府和市委的正确领导下，在市人大、市政协的监督、支持下，市政府组织和带领全市人民进一步抢抓机遇，克服经济下行压力影响，攻坚克难，开拓进取，圆满完成了各项目标和任务，经济社会呈现出良好发展态势。

　　(一)经济加快发展，综合实力持续增强

　　主要经济指标快速增长。2012年，地区生产总值预计完成1022亿元，首次突破1000亿元，比上年增长15%，比2007年增长1.4倍;规模以上工业增加值385亿元，比上年增长18%，比2007年增长2.2倍;公共财政预算收入实际完成127.6亿元，比上年增长23%，比2007年增长2.6倍;全社会固定资产投资预计完成920亿元，比上年增长30%，比2007年增长2.9倍，五年累计完成3146亿元;社会消费品零售总额380亿元，比上年增长17%，比2007年增长1.4倍;实际利用外商直接投资12亿美元，比上年增长18%，比2007年增长7.2倍，五年累计完成37.3亿美元;外贸出口总额28亿美元，比上年增长18%，比2007年增长73%;引进内资项目到位资金810亿元，比上年增长31.9%，比2007年增长9.7倍;城镇居民人均可支配收入19555元，比上年增长14.2%，比2007年增长99%;农民人均纯收入11640元，比上年增长16%，比2007年增长1.1倍。

　　工业经济不断壮大。通过实施老工业基地振兴战略，工业经济占全市经济比重由2007年的37.6%提高到43.6%。2012年底，全市规模以上企业850户，主营业务收入预计完成1280亿元，比2007年增长2.5倍。其中亿元以上企业310户，有2户企业成长为百亿元企业，骨干企业对工业经济的牵动力不断增强。工业投入五年累计完成1600多亿元，比前一个五年翻了三番。本钢不锈钢冷轧、丹东港大豆深加工、哈曼音响等一批工业大项目成功引进并实现竣工投产，黄海新能源汽车等项目开工建设。技术创新能力进一步增强，2012年规模以上企业高新技术产品增加值102亿元，无水印染等11项科技成果达到国际领先水平，截至目前，拥有“欣泰”等6枚中国驰名商标。汽车及零部件等支柱产业、仪器仪表等优势产业、新能源等新兴产业业已确立，2009年以来启动建设的辽宁(丹东)仪器仪表产业基地、环保产业园、再生资源园等重点工业园区已初具规模且发展势头良好。2012年，面对工业经济下行压力，出台并实施了一系列扶持政策，促进了工业经济企稳回升。

　　农村及县域经济快速发展。五年来，农业全面丰收，粮食产量2011年创99.6万吨历史新高后，2012年达到95.2万吨，成为第二个高产年。东港、宽甸分别被省政府确定为草莓、人参“一县一业”示范县。东港梭子蟹等8种农产品获得国家地理标志认证。坚持科技兴农，建设优质稻米等9个特色农产品基地，培育了丹东蓝莓等10个特色品牌。设施种植业、设施渔业、畜禽标准化养殖小区快速发展。农业出口创汇能力不断增强，2012年农产品出口额预计达到6.9亿美元，增长20%，出口增量位居全省首位。农村水利、生态建设加快实施，五年投入农田基本建设资金约30亿元，有望连续四年夺得省政府农建“大禹杯”，“青山工程”取得阶段性成果。连续三年经受住了特大洪水袭击和强台风威胁的考验。农村扶贫和海洋环保工作走在全省前列，强农惠农富农政策有效落实，伏季休渔和渔船管控得到加强。县域经济综合实力明显提升，县域超百亿元产业集群达到2个，佰山木业等5个10亿元以上农业产业化大项目开工建设，东港市、凤城市跻身全国百强县行列。5个边境县(市、区)均被列为全国兴边富民行动重点县。

　　城区经济和服务业发展水平持续提升。服务业增加值五年翻了一番多，连续两年超过地区生产总值增长速度。商业流通服务网络覆盖城乡，锦山中央商贸商务服务业集聚区等14个重点集聚区已形成规模，万达广场等城市综合体项目相继开工建设，沃尔玛等国际连锁商业企业进驻丹东，皇冠假日等五星级宾馆投入使用，极大提升了我市服务业的档次和水平。会展业逐步培育起来，旅游节、台湾周、仪器仪表博览会的成功举办扩大、提高了丹东的影响力和知名度，特别是去年举办的首届中朝经贸文化旅游博览会进一步巩固了丹东对朝鲜各项合作交流的重要地位。旅游业发展迅猛，假日经济持续火爆，天桥沟、凤凰山等精品景区建设已见成效，荣获了中国“温泉之城”、“十大宜居中小城市”、“十大养老胜地”等称号，丹东已经成为辽宁重要旅游城市和赴朝旅游集聚区。房地产业保持了健康发展的态势。城区经济完成了第一个“三年倍增”计划，通过向城区下放管理权限等举措，城区发展潜力逐步释放出来。

　　财政金融支持发展能力进一步提高。公共财政预算收入五年累计完成410亿元，税收累计完成300亿元，收入质量稳步提高。财政支出结构趋于合理，用于农业、教育、科技等民生方面支出增长超过20%。有效盘活政府资产，建立融资平台，成功发行两期城投债券，支持了城市建设。2012年，全市本外币各项存款余额1245亿元，比上年增长17%;贷款余额682亿元，比上年增长18%，新增贷款连续三年突破百亿元。欣泰电气上市获得中国证监会审核通过，即将上市。五年引进和组建了近60家金融机构。

　　(二)对外开放实现重大突破，各项改革不断加快

　　沿江沿海重点区域开发开放成效显著。抓住2009年辽宁沿海经济带上升为国家战略的重大机遇，一批对全市经济社会发展有深远影响和牵动力的重大项目得以实施，五年新开工亿元以上项目545个。城市发展空间有效拓展，实现丹东新区、东港、大孤山多点布局，点线面联动发展。丹东新区迅速崛起，累计实施了市民服务中心、丹东二中、体育馆等城市功能项目21个，科技五金城、西湖城农贸大市场等商贸地产项目53个，7个重点产业园区初具规模，九纵九横主干路网等基础设施基本完成，新区正在从建设开发向管理职能提升转变。大孤山经济区总体发展规划通过省专家组评审，湿地调整方案获国务院批准，港口、疏港路、精密加工园区等各项建设全面铺开。启动了翡翠湾开发，香港佳兆业房地产项目率先开工，标志着新老城区开始对接建设。中朝共同开发和共同管理黄金坪经济区实现突破，中方配套区启动建设并正在积极推进。

　　外向型经济实现新跨越。与100多个国家和地区实现了经贸往来，五年完成外贸出口总额近100亿美元，出口商品结构趋于优化，对朝贸易集散地地位日益显现。招商引资成效显著，利用外资连续两年突破10亿美元，引资质量有效提高，生产型、高技术型、出口创汇型项目占80%以上，与哈曼、SK、希尔顿等国际大公司实现了合作。境外投资领域不断拓宽，思凯电子、曙光集团等11户企业实现了海外并购。东北东部(12+2)区域合作纳入《东北振兴“十二五”规划》和国家发改委“十二五”重点区域发展战略，城市间经贸、文化、旅游合作进一步深入。

　　各项改革有序推进。政府机构新一轮改革任务顺利完成，行政审批制度改革取得阶段性成果，新整合的公共资源交易中心挂牌服务。全市90%以上的事业单位推行了聘用制。财政体制改革进一步深化。政府办基层医疗卫生服务机构全面实行基本药物制度。林权制度改革全面完成。

　　(三)基础设施日臻完善，城市面貌发生巨大变化

　　城市环境焕然一新。启动并积极开展“四城联创”活动，获得国家“园林城市”和省级“卫生城市”、“环保模范城”称号，在全省城市文明指数测评中获得第二名。城市规划更注重于现代化、人性化理念。人均占有绿地面积10.6平方米，比2007年增加4.3平方米，城市绿化美化亮化档次和品位不断提升。改造完成并免费开放了城市三大公园。积极实施“蓝天工程”，仅去年推行城市集中供热就废弃烟囱近百个，空气质量优良天数达到345天以上。完成了国家“十一五”主要污染物减排任务，淘汰关闭污染企业900多家，建成并运行城镇污水处理厂5座，鸭绿江下游水质明显改善。

　　基础设施更加完备。蒲石河抽水蓄能电站、金山热电等重大电力设施项目实现并网发电，三湾水利枢纽土建主体工程竣工。东北东部铁路建成通车，沈丹客专、丹大快速铁路建设加快推进。丹海、丹通高速公路全线通车。实施了一号、三号干线贯通，二号干线续建，四号干线启动建设等道路工程。中朝两国合力推进的新鸭绿江公路大桥主桥、引桥施工全面展开。丹东港五年投资300多亿元，18个泊位建成投入使用，20万吨级矿石泊位开工建设;2012年吞吐量达到9600万吨，比2007年增长2.7倍，跃居全国港口第14位。规划中的另一个亿吨大港——海洋红港区实现开工。丹东机场改扩建工程基本完工。如此密集的基础设施建设，在全国同等规模城市中极为少有。

　　(四)民生工作得到加强，各项社会事业进一步繁荣

　　民生工程惠及城乡。五年来，用于民生工程的财政支出增量占全部财政支出增量的80%，解决了一批百姓关注的难点、热点问题，各项惠民实事如期完成。对城市近200条街路进行大修改造，实施了120多条群众最关心的小巷路改造工程，更换了100台环保公交车，极大改善了群众出行条件。仅去年就封堵垃圾通道4000多个，新投放垃圾收集车70多台，增加果皮箱6000多个，解决了困扰百姓生活几十年的二次污染问题。五年建设廉租房、公租房、经济适用房及棚户区改造住房4.4万套230万平方米。下大气力解决供暖问题，仅最近两年，市财政就拿出1.5亿元用于供暖保障，特别是2012年实施了550万平方米7万户金山热电集中供热联网改造工程，供暖质量进一步提高。基本养老等5项社会保险扩面均超额完成省下达任务，社会保障体系框架基本形成，城乡低保户救助、新农合补助标准不断提高。五年累计实现实名制就业32.3万人次。

　　社会事业全面发展。完成了义务教育“双高”普九任务，建成农村九年一贯制(寄宿制)学校60所，新二中、新四中、新职专投入使用，提前完成了74万平方米的校舍安全三年改造任务。初步实现了城乡基本医疗卫生全覆盖，完成了一院、三院、中医院、县级医院等医院改扩建工程。食品药品安全保障工作不断加强。低生育水平继续保持。公共文化服务体系基本建成，丹东大剧院、新图书馆等文化设施开工建设，博物馆、图书馆、文化馆免费对外开放。全民健身蔚然成风，连续承办鸭绿江国际马拉松赛、中国女篮甲级联赛等国家级品牌赛事，百公里山水相连健身步道部分路段建成。实施了“百千万”人才引进工程和高端人才引进活动，设立院士工作站10个，国家级博士后科研工作站和省级博士后科研基地15个，引进海外研发团队项目37个。耕地、矿山、地下水等各类自然资源得到了合理保护和利用。实现了全国双拥模范城“六连冠”和全省“七连冠”，国防教育和民兵预备役等工作取得新成果。

　　民主法制建设不断加强。坚持向人大常委会报告和向市政协常委会通报工作情况，自觉接受人大依法监督和政协民主监督，认真听取各民主党派、人民团体和各个方面的意见。五年共办理市人大代表建议、政协提案2863件，办结率100%，满意率99.9%。依法行政扎实推进，普法教育有效实施。政务公开稳步向基层延伸，基层政权建设进一步加强，村委会和社区居委会办公、活动场所条件明显改善。审计监督职能得到发挥。农村集体资金、资产、资源委托代理全面铺开，“五大系统”建设深入开展，在全省率先实行行政问责，严厉查处了损害群众利益的违法违纪问题。加强和创新社会管理，着力排查、化解信访积案和各类社会矛盾，社会管理综合治理得到加强，应急管理体系进一步完善，安全生产形势总体平稳，边海防管理水平有效提升，保持了边境地区的和谐稳定。

　　外事、口岸、侨务、台务、民族、宗教、统计、物价、保密、档案、接待、地方志、人防、气象、防震减灾、妇女儿童、老龄、残疾人等工作也取得了新成绩。

　　各位代表，本届政府正值辽宁沿海经济带全面开发建设的重要阶段。五年来，我们牢牢抓住东北老工业基地全面振兴和辽宁沿海经济带上升为国家战略的双重机遇，逐步实现了发展重点由江向海的转身，发展方式向高质量、快速度的转变，发展成果向更多惠及人民群众的方向集聚。这些成就是在市委的正确领导和市人大、市政协的监督、支持下取得的，是在全市人民的共同努力下取得的。在本届政府任期届满之际，我代表市政府向全市各族人民，向各民主党派、各人民团体和各界人士，向驻丹中省直单位和驻丹部队、武警官兵表示衷心的感谢并致以崇高的敬意!

　　在肯定成绩的同时，我们也清醒地认识到，全市经济社会发展还存在一些亟待解决的问题，突出表现在：一是经济总量偏小的状况没有得到改观，在全省仍处于靠后位置;二是工业主导产业不突出，产业集聚不明显，产业类型分散，工业项目引进、落地数量少，特别是缺少工业大项目，难以形成较强牵动力;三是现代服务业发展与沿海发达地区比还有很大差距，潜力和优势没有充分发挥出来;四是财政困难状况没有得到有效缓解，债务负担较重，致使社会保障、城市建设资金压力巨大;五是一些部门和干部作风不实，庸懒散现象依然存在，投资软环境还需进一步改善。这些问题已引起我们的高度重视，在今后的发展中，将采取切实有效的措施加以解决。

　　二、今后五年发展思路和奋斗目标

　　今后五年，是深入贯彻党的十八大精神，全面建成小康社会的重要时期，也是完成“十二五”规划目标任务的关键时期。我市面临着丹东沿江沿海经济全面发展，黄金坪合作开发实质推进，新鸭绿江公路大桥、沈丹客专、丹大快速铁路等一批重大基础设施项目竣工并集中发挥作用等机遇，也面临着现实困难与历史遗留问题交织凸显等压力。因此，惟有抢抓机遇、开拓创新、破解难题，才能保持经济的稳步健康发展;惟有心无旁骛、少说多做、狠抓落实，才能把发展成果更多地惠及民生。为此，今后五年政府工作的总体思路是：高举中国特色社会主义伟大旗帜，以邓小平理论、“三个代表”重要思想和科学发展观为指导，深入贯彻落实党的十八大精神，全面落实市第十一次党代会和市委十一届五次全会各项部署，紧紧围绕率先全面建成小康社会的总目标，以发展为第一要务，以开放为主基调，以创新为驱动，以保障和改善民生为着眼点，坚持高质量、快速度发展理念，全力开发北黄海，切实转变经济发展方式，加快促进产业升级，持续壮大地区综合实力，把发展成果更多更公平惠及人民，建设文明、生态、繁荣、富裕的国际化港口城市，努力构建大气秀美新丹东。

　　依据上述总体思路，今后五年政府工作的主要奋斗目标是：

　　——综合经济实力稳步提升。地区生产总值年均增长12%以上;公共财政预算收入年均增长15%以上;全社会固定资产投资年均增长20%以上。经济发展的基础更加牢固，发展方式转变明显，发展动力不断增强。

　　——开放空间有效拓展。着力打造“一桥两岛”开放新格局，推动丹东新老城区、黄金坪配套区、前阳开发区、东港市区、大孤山经济区一体化发展，形成城市带、产业群;利用外资规模不断扩大，形成以技术、品牌、质量为核心的外贸出口竞争新优势。海洋经济开始崛起，港口牵动作用进一步增强，丹东港吞吐量力争达到2亿吨，海洋红港区吞吐量超过2500万吨，成为东北东部最便捷的出海通道。

　　——产业结构合理优化。农业基础性地位进一步巩固;工业发展水平明显提升，具有丹东特色的工业体系基本形成，战略性新兴产业和先进制造业迅速壮大;信息、物流、会展、金融等现代服务业在经济结构中的比重大幅提高。

　　——生态环境极大改善。资源节约型、环境友好型社会建设取得重大进展，可持续发展基础进一步牢固，以“蓝天、碧水、青山”工程为依托，着力推进绿色发展、循环发展、低碳发展，地区生产总值能耗及主要污染物排放大幅下降，国家级生态市建设各项指标达到创建标准，生态、宜居的丹东激发出新的魅力。

　　——人民生活水平全面提高。统筹推进城乡社会保障体系建设，实现更高质量的就业。城镇化率达到70%以上。城乡居民收入力争实现翻番，居住条件和环境进一步改善，民生问题得到妥善解决，社会各项事业协调发展，社会安定和谐，群众安居乐业。

　　为实现这些目标，我们将在八个方面付出更大的努力：一是以发展海洋经济为引擎，形成北黄海开发新增长极。二是以提升企业科技创新能力及核心竞争力为着力点，塑造丹东工业新辉煌。三是以打通东北东部出海大通道为契机，助推服务业新飞跃。四是以产业集群建设为带动，促进县域经济实力再上新台阶。五是以高水平建设新兴区域为重点，迈出城市建设管理新步伐。六是以天蓝地绿水净为标志，走出可持续发展新路子。七是以发展成果惠及民生为目标，实现人民群众生活水平新提高。八是以为民服务为宗旨，树立政府自身建设新形象。

　　三、2013年全市经济和社会发展主要任务

　　2013年是新一届政府履职的第一年，面对严峻的经济形势，我们既要居安思危、未雨绸缪，又要坚定信心、不畏艰险，把稳增长放在更加重要的位置，把丹东的经济社会发展好，把人民群众的事情办好。为此，我们确定今年全市经济和社会发展的主要目标是：地区生产总值增长12%;规模工业增加值增长13%;公共财政预算收入增长15%;全社会固定资产投资增长20%;社会消费品零售总额增长15%;实际利用外商直接投资10亿美元;外贸出口增长10%;引进内资项目到位资金增长20%;城镇居民人均可支配收入增长13%;农民人均纯收入增长14%。为实现上述目标，重点做好以下九项工作：

　　(一)全力抓好项目工作

　　把招商引资做强。强化主题招商，围绕大孤山等重点区域，精细化工等重点产业，积极引进对我市产业结构调整有促进作用的优质项目。强化节会招商，通过举办中朝经贸文化旅游博览会等国内外关注的大型展会活动，增强丹东吸引力。强化重点区域招商，密切与日韩、香港、台湾、京津冀等国家和地区的联系，做好对总部或区域总部的招商，加强与东北东部乃至东北地区腹地的合作交流。强化以商招商，加大产业链和产业集群招商力度，带动产业链上下游项目入驻。

　　把项目落地做实。切实提高环评、规划等项目审批效率，注重节约用地，做好土地征用、动迁、收储和招拍挂等工作，提高项目投资强度。抓好项目的跟踪落实，积极主动地帮助投资者解决项目建设中的各类问题，确保项目尽快竣工达产。确定三月、四月为项目开工月，确保再新开工一批有影响力的项目。

　　把项目储备做精。围绕环境保护、民生改善和社会发展，筛选、包装、储备一批重大基础设施项目;围绕重点园区、重点产业集群，筛选、包装、储备一批技术含量高、牵动力强、投资回报率大的产业发展项目，促进项目储备的常态化、系统化。

　　(二)进一步加快以北黄海和沿江沿海经济带开发为重点的对外开放步伐

　　坚定不移地加快新区发展。切实提高新区管理水平，促进新区向新城转变。以汇聚人流、物流，提升人气、商气为重点，采取市场化运作等方式，进一步完善新区城市功能和基础设施建设。居然之家等商业设施加快实施，新区医院尽快启动，体育场、大剧院等文体设施加快推进，不断完善新区、繁荣新区。

　　快速推进大孤山经济区建设。做好临港产业园、精密加工园、东疏港路等基础设施建设，确保红星开关、方元铸造、锦鸿数控等精密加工园入驻企业年底前竣工达产。加快重化工业招商引资和发展，重点推进翔鹭腾龙集团炼化一体化等大项目建设。

　　全面实施翡翠湾开发。加快区域内基础设施建设特别是路网建设，通过招商推介、媒体网站宣传等方式，不断扩大翡翠湾的知名度。以打造现代服务业集聚区为重点，有针对性地开展招商引资，跟踪服务好佳兆业等开工项目，争取新东方商贸城、中国供销集团农商物流港等项目开工建设。

　　积极开展黄金坪中方配套区建设。争取黄金坪中方配套区尽快获得国家批准，并得到国家综合保税区等相关优惠政策支持，尽快与黄金坪形成全面对接条件，全力推进恒力纺织工业园等项目建设。力争使我市年内进入国家第二批沿边地区开发开放试验区行列，高起点做好规划和产业布局。

　　进一步推动港航经济发展。加快建设丹东港20万吨级矿石泊位等港口基础设施，实现亿吨大港目标;积极推进丹东港与韩国大宇、现代，美国史密斯菲尔德等世界500强企业的合作。抓好海洋红港区防波堤、围堰、航道等工程实施，启动5万吨级泊位建设。进一步做大做强临港工业，拉长临港产业链条。大力发展港航物流和第三方、第四方物流。加快推进新鸭绿江公路大桥及口岸区、口岸物流园区、安民口岸区建设，带动口岸经济发展。高质量编制《东北东部经济带发展规划》，推动和引领东北东部(12+2)区域合作务实深入开展。

　　大力发展外向型经济。加强出口基地建设，积极培育出口品牌和新的出口增长点，不断扩大对外合作领域和贸易规模，特别是进一步加强对朝合作交流、经贸往来。鼓励和支持我市企业走出去开拓国际市场，并购和引进国外科技型企业及先进技术。

　　(三)不断增强工业经济支撑和拉动作用

　　保持工业回升态势。建立和完善重点企业运行监测、用电情况分析和生产要素保障系统，落实各项扶持政策，全力支持企业抓订单、找市场、融资金。促进有潜力的停产半停产企业全面恢复生产;积极引进和落实工业项目，特别是工业大项目;千方百计破解难题，加快在建项目投产达产，形成新的增长点。

　　壮大工业企业规模。采取改造、并购、重组、引进、上市等措施，积极推进百户骨干企业提升、百户亿元企业培育、百户小微企业成长“三百企业工程”的实施，促进现有工业企业发展壮大，确保年内新增10亿元以上企业2户、规模以上企业40户。

　　做强产业集群。全力抓好汽车及零部件、仪器仪表和再生资源3个省级产业集群，力争使防护纺织制品、增压器2个专业产业集群跨入省级行列。促进电子信息及软件外包、纳米印刷制版等产业成长壮大，推动精细化工产业升级，逐步打造“大孤山石油炼化和精深加工一体化、宽甸凤城硼镁化工一体化和无水印染技术产业一体化”三大精细化工产业基地。抓好工业园区建设，进一步完善考核、评价、激励机制和公共服务平台体系。

　　提高企业自主创新能力。加强产学研合作，推进企业研发机构和平台建设，抓好重点领域技术创新和科技攻关，密切与中科院等科研院所的合作，提高科研成果转化能力，加快新技术新产品新工艺研发应用，提升企业综合竞争力。确保高新技术产业园区获得省级批准，大力实施创新型和产业化项目，促进科技型企业向高新园区聚集。

　　(四)继续保持县域经济快速发展

　　切实提升县域经济实力。完善县域产业园区基础设施和配套，重点抓好东港再生资源、凤城紧固件、宽甸非金属新型材料等县域重点产业集群建设，推动这些产业集群向百亿元规模迈进。争取实现10亿元以上农业产业化大项目全部竣工投产。抓好“一县一业”，不断提高东港草莓、宽甸人参和凤城板栗产业化发展水平，积极培育龙头企业。

　　加快发展现代农业。加强耕地特别是基本农田保护，强化农业科技创新和推广，稳定粮食生产，提高种粮效益。加大农产品质量安全体系建设，加强农民合作经济组织建设，推进农业标准化生产，增加标准化生产面积10万亩，规模化畜禽养殖比重达到62%以上。抓好现代渔业示范小区建设，水产品总产量达到60万吨。着力推进设施农业、订单农业发展，努力实现农产品出口创汇增长15%以上。

　　积极稳妥推进城镇化建设。对丹东市、县城、小城镇进行科学规划、合理布局，有效提高容纳力和承载力。加快城市东部、北部地区开发建设，推动县城向中等城市发展，完善小城镇基础设施，鼓励和支持有条件的农村人口向小城镇聚集，着力提高城镇化质量。

　　不断改善农村生产生活条件。开展农村土地综合整治，加强农村基础设施建设，积极实施绿化造林，加快推进“青山工程”。抓好农村水利建设，实施大洋河等河流综合整治，深入开展农建“大禹杯”竞赛活动。进一步加大在科技、金融、医疗等方面对“三农”的服务力度，提倡开发式扶贫，着力促进农民增收。

　　(五)加快推进现代服务业发展

　　促进城区经济繁荣活跃。城区经济重点抓好服务业发展，万达广场等城市综合体项目争取部分交付使用，尽快形成高端服务业集聚区。加快实施新安步行街地下商城、华美新天地、天赐国际金融中心和希尔顿、喜来登酒店等商业项目。积极发展会展、研发设计、文化创意等产业，打造一批新的服务业集聚区，推动老商业区实现现代服务业升级改造，抓好商业市场建设，培育发展好“夜经济”，保持房地产业健康发展，培育一批拉动力强的消费增长点。

　　推动旅游业做大做强。实施精品旅游战略，丰富提升旅游文化内涵，做大做强有地方特色的旅游产品，陆续推出具有丹东民俗风情和地域特色的常态化演出节目。争创鸭绿江国家5A级旅游景区及创建国家级赴朝旅游集聚区。发展沟域经济，实施生态立县，着力把宽甸打造成“辽宁生态旅游第一县”。尽快完成《全市温泉旅游产业发展规划》的编制和评审，进一步整合温泉资源，积极引进和实施高档次的温泉旅游项目。大力开发观光农业、观光工业，鼓励发展“农家乐”，不断提高旅游整体接待能力。加大旅游宣传、促销和区域合作力度，拓宽旅游客源市场。

　　(六)积极发挥财政金融支撑作用

　　提高财政保障能力。不断壮大现有税源，培育后续税源，提高税收占公共财政的比重，增强财政可支配能力。强化部门预算约束，推进财政支出绩效评价，严格控制“三公”经费，建立厉行节约的长效机制。加强财政监督检查，规范财政收支秩序。

　　强化金融支持作用。积极争取金融支持、信贷授信，切实缩小存贷差，有针对性地开展政银企项目对接。尤其加大对小微企业的信贷支持力度。拓宽融资渠道，支持、培育企业上市，发挥小额贷款公司、担保公司、村镇银行等新型金融机构融资作用。力争全年实现各类融资220亿元以上。

　　防范财政金融风险。规范资金筹集，强化支出约束，科学合理控制债务规模，加强对政府债务举借、使用和偿还的全过程管理，有效防范政府债务冲击。强化国有资产监管，确保国有资产保值增值。不断改善金融生态环境，降低各商业银行不良贷款率，坚决打击恶意逃废债行为。

　　(七)切实保障和改善民生

　　今年将全力抓好与群众生活息息相关的各项民生工作，让群众生活得更加舒心、幸福。

　　1、实施菜篮子工程。抓好不低于30万亩的菜篮子基地建设，进一步增加本地肉蛋奶、蔬菜的品种和产量;规划建设好城区农贸市场，有效改善现有农贸市场购物环境;加强市场管理，严厉打击欺行霸市行为，扩大肉蛋奶和蔬菜直销覆盖面，着力解决农民卖菜难和居民买菜贵的问题。

　　2、实施城乡饮水安全工程。分期对城区近300座自来水二次加压泵站及分支管网进行安全用水改造;解决好农村11万以上人口饮水安全问题。

　　3、实施百姓健康工程。市中心医院外科病房楼全面竣工;在全市范围内实施免费孕前优生健康检查;对群众购买的假劣药品实行“先行退付制度”，在社区开展家庭过期药品兑换活动，保障群众用药安全。实施万隆体育场等群众健身场地改造，在城乡投放100套健身器材，建设一批免费开放的电子阅览室，让群众享受健康的文化、体育生活。

　　4、实施群众温暖工程。建设保障性住房1050套，实施棚户区改造24万平方米;继续推进金山热电集中供热联网工程，不断提高供暖服务质量。

　　5、实施老旧小区改造工程。逐步改造、维修、更换城市老旧小区居民楼燃气、上下水、暖气、用电线路等设施;继续实施街巷路及延伸至小区内道路改造维修的“小巷工程”，有计划地解决弃管楼问题。

　　6、实施城市畅通工程。改造、拓宽人民街、金海路、青年大街等重要路段;打通春四路与沿江路、新城区与201国道的连接路;实施主城区铁路与公路平交路口改造，拓宽国桢路与铁路隧道交叉路面，建设春五路与铁路交叉人行地道;实施城区部分道路大修及北桥洞等桥梁维修工程;在花园立交桥等交通事故易发路段设置安全隔离带;增加新老城区公交车次，年内实现主要公交线路延长至新区;在城市人口密集和商业集聚区域启动建设1000个以上停车位，规划部分路段实行限时停车。

　　7、实施就业和社保体系完善工程。不断拓宽就业渠道，鼓励以创业带动就业，全年实现实名制就业6万人，城镇登记失业率控制在4.2%以内;进一步扩大养老、医疗、失业、工伤、生育等社会保险覆盖面，将城镇各类新增企业及职工纳入社会保险范围，认真落实失地农民养老保险政策。

　　8、实施特殊困难群众关爱工程。全额资助城乡近10万名低保对象参加城镇居民医疗保险和新农合;为困难家庭提供减免收费的法律援助;对幼儿园中接受学前3年教育的经济困难家庭儿童、孤儿和残疾儿童实施资助;对100个社区办公场所及残疾人活动场所实施无障碍改造;扶持54所民办养老机构，改善服务设施，提升服务水平。

　　(八)大力加强社会建设

　　促进各项社会事业协调发展。加大公办幼儿园建设力度，推进基础教育强县(市、区)建设，加快发展现代职业教育，积极发展继续教育。推进公共卫生服务均等化，进一步提高新农合筹资标准，开展大病医疗保障统筹，推行县级公立医院改革，适时扩大基本药物制度实施范围。不断提高出生人口素质，积极应对人口老龄化。完善公共文化服务体系，大力发展文化产业，不断深化文化体制改革。全面落实《全民健身计划》，做好十二届全运会丹东赛区各项工作，筹备好2014年在我市举行的十二届省运会。实现丹东广播电视节目在农村地区的全覆盖。继续实施“鸭绿江人才引进计划‘311’工程”，着力引进一批高层次创新型人才。鼓励全社会积极投身慈善事业。加强民兵预备役建设，抓好全民国防教育和双拥共建，实施好在我市开展的国家边海防基础设施示范区建设和市级国防动员信息化建设试点工作。

　　加强生态文明建设。大力推进“四城联创”活动，深入开展“文明使者在行动”，努力构建和谐的人际关系，进一步激发广大群众共建文明城的热情。做好国家级生态市创建的各项准备工作。实施地下水和空气负氧离子监测，完成160家重点企业清洁生产强制审核。抓好城市绿化亮化美化，继续开展内河整治。强化对城市交通、市场经营、环境卫生等方面的管理。实施农村环境连片整治，继续开展生态村镇创建活动。

　　保持社会和谐稳定。深入开展“六五”普法宣传教育。加强和创新社会管理，开展社会管理综合治理，严厉打击传销和制假售假行为，加大边海防管理力度，保持对走私、贩毒、越界捕捞等违法犯罪打击的高压态势。做好民族宗教工作，继续开展兴边富民行动。增强国家安全意识，保持边境地区稳定。加强基层政权和社区建设，确保村委会和社区居委会换届选举顺利完成。加强应急队伍建设，提高城乡减灾能力。切实抓好安全生产工作，坚决遏制较大以上安全生产事故的发生。全力维护劳动者合法权益。深入推进领导干部接访下访和信访积案攻坚，全面落实社会稳定风险评估和矛盾纠纷排查化解机制。加强对食品药品安全、重大动物疫情防控和市场价格的监管，营造安全放心的消费环境。

　　(九)努力建设高效务实责任政府

　　加强依法行政。围绕法治政府建设，进一步规范行政决策程序，把公众参与、专家论证、风险评估、合法性审查和集体讨论作为重大决策的必经程序。加强绩效管理和考核。规范行政执法，强化行政复议。推进财政预算、公共资源配置、重大项目建设等信息公开，让老百姓拥有更多知情权、参与权、表达权、监督权。自觉接受人大法律监督和政协民主监督，切实做好人大代表建议和政协提案办理工作，不断提高政府公信力和执行力。

　　改进工作作风。坚持少说多做抓落实，注重到群众中倾听民声、解决问题。市政府领导带头做到：经常深入基层调研、现场办公;到基层调研、检查工作不用警车开道，不迎送，随行部门一般不超过3个，在市区调研不就餐，到县(市)调研吃工作餐;严格控制以市政府名义召开的各类会议，一般性会议不超过1小时，领导讲话一般不超过15分钟;各级汇报会、座谈会不悬挂欢迎横幅，不摆鲜花、水果、香烟等;严格控制文件简报数量和发放范围;经省、市政府批准的外出公务等活动，严格控制随行人数;不参加下属单位或企业安排的宴请和高消费娱乐活动。政府部门之间不搞相互宴请。坚决治理庸懒散、效能低下、工作不作为等现象，切实提高行政效率，让群众、企业办事更加通畅和便捷，在全市形成良好的服务软环境。

　　注重廉政建设。进一步完善惩防体系和“五大系统”建设，强化行政监察和审计，做好对改进工作作风的监督落实，严格行政问责。深化行政审批制度改革，进一步完善公共资源交易系统，加强对行政权力运行、财政资金使用、经济责任履行等领域的监督，强化对农村集体资金、资产、资源的管理，全面推动政府廉政建设，树立勤政、廉洁的政府形象。

　　各位代表，新一届政府面临着艰巨而繁重的任务，担负着光荣而神圣的使命。让我们在党的十八大精神指引下，在市委的正确领导下，在市人大、市政协的监督、支持下，团结带领全市各族人民万众一心，开拓进取，埋头苦干，扎实工作，不断推进大气秀美新丹东建设，为率先全面建成小康社会而努力奋斗!
